# The mechanisms of planar cell polarity, growth and the Hippo pathway: Some known unknowns

**DOI:** 10.1016/j.ydbio.2013.01.030

**Published:** 2013-05-01

**Authors:** Peter A. Lawrence, José Casal

**Affiliations:** Department of Zoology, University of Cambridge, Downing Street, Cambridge CB2 3 EJ, United Kingdom

**Keywords:** Planar cell polarity, Growth, Gradients, Morphogens, Hippo, Fat, Dachsous, Wnt, Frizzled, Van Gogh, Starry night

## Abstract

Planar cell polarity (PCP) is a small but important area of research. In this review we discuss a limited number of topics within the PCP field, chosen because they are difficult, unsolved, controversial or just because we find them interesting. Because *Drosophila* is the best studied and technically most amenable system we have concentrated on it, but also consider some examples from work on vertebrates. Topics discussed include the number of genetic pathways involved in PCP, as well as the causal relationship between embryonic axes, gradients of morphogens and PCP itself. We consider the vexed question of the roles of the Wnt genes in PCP in both vertebrates and *Drosophila*. We discuss whether the proteins involved in PCP need to be localised asymmetrically in cells in order to function. We criticise the way the Hippo pathway is described in the literature and ask what its wildtype function is. We explore afresh how the Hippo pathway might be linked both to growth and to PCP through the gigantic cadherin molecule Fat. We offer some new ways of making sense of published results, particularly those relating to the Frizzled/Starry night and Dachsous/Fat systems of PCP.

## Highlights

► We review the number of genetic pathways involved in PCP. ► The causal relationship between embryonic axes, morphogen gradients and PCP. ► The asymmetric localization of proteins involved in PCP. ► The roles of the Wnt genes in PCP in both vertebrates and *Drosophila*. ► The function of the Hippo pathway and its link to Fat.

“There are known knowns; there are things we know we know. There are known unknowns; we know there are some things we do not know. There are also unknown unknowns; we don’t know we don’t know.”

Donald Rumsfeld (United States Secretary of Defense), February 12th 2002.

## Introduction

Planar cell polarity (PCP) refers to the polarity of a cell *within* the plane of an epithelium (Nübler-Jung et al., 1987); it is different from apico-basal polarity both conceptually and mechanistically. PCP is an over-reviewed subject; the many reviews mostly rehash the same experimental findings, testing the patience of the reader (for a comprehensive list of recent reviews see Yang, 2012). Our aim is to test the patience of the reader in an alternative way; in reviewing PCP we emphasise uncertainties which have been forgotten or ignored. We also discuss the relationship between PCP and growth, a topic that resembles a minefield.

Over the last 100 years or more, embryologists have concentrated on how cells know their place in the embryo, on how such positional information (Wolpert, 1996) is conveyed and interpreted to determine a cell's identity as well as the fates of its daughter cells. Positional information is usually encoded in a pervasive gradient, the concentration of a morphogen at each locale giving scalar information to the cells (Lawrence, 2001a). But identified cells in embryos also need to move in one particular direction or send an axon in one direction or divide and migrate to grow preferentially in one direction. Thus, to build an animal properly, embryonic cells must have access also to vectorial information. This vectorial information can be directly and simply expressed in the orientation of subcellular and/or multicellular structures such as stereocilia in the inner ear, bristles on a fly or mammalian hairs (Goodrich and Strutt, 2011). But orienting a cell is not simple and depends on diverse inputs and processes — a hidden complexity that has lead to confusion and disagreement amongst experts.

During the history of embryology few scientists have studied PCP, and this is largely because of the dictates of fashion, but also because research into PCP has proved difficult. Why? One reason is that PCP is a contextual phenomenon — what matters is the alignment of a cell with respect to the axis of an appendage (distal or proximal?) or of an embryo (anterior or posterior, dorsal or ventral?). Thus PCP needs to be studied in context, *in situ* and *in vivo* and these can be demanding requirements. Also there is another hindrance, studies of PCP have been limited because, although some cells make conspicuous and oriented outgrowths, the polarity of most cells is concealed. This difficulty can sometimes be overcome: noone had seen PCP in the *Drosophila* blastoderm and yet, if one protein, Slam, is artificially over-expressed at that early embryonic stage, these apparently unpolarised cells place Slam along the antero-posterior axis of the cell (Lecuit et al., 2002; Zallen and Wieschaus, 2004) suggesting that components of a PCP machinery are present and active. Nevertheless, PCP has been mostly investigated in systems in which the polarity of each cell (or group of cells) is signalled by oriented structures. This restriction of itself is benign, but it can foster the dubious assumption that plain epithelial cells, those that have no outgrowths, are unpolarised. The number of developmental phenomena recognised as depending on PCP has increased massively in recent years. The phenomena include cell migration, as in convergent extension and in neurulation, neurogenesis, axonal guidance, dendritic branching, kidney morphogenesis and vasculogenesis (Wang and Nathans, 2007; Gao, 2012).

It is not yet clear whether the basic mechanisms of PCP are universal, although this is argued by the conservation of the main genes from flies to mammals. But, in any case, it always makes sense to focus research on the most convenient system. For PCP there is no doubt this system is *Drosophila* and the reasons are mainly technical. *Drosophila* of course has plenty of genetics but also has tissues consisting of simple monolayers of cells, with each cell displaying its polarity in cuticular structures. Also, no system has better methods of marking genetic mosaics, cell by cell. For these reasons we will concentrate here on flies, with short excursions to mammals.

## Operational approach to the mechanisms of PCP

Cell interaction is at the heart of PCP. Cells are polarised in response to information coming from other cells: this can be of two kinds. There can be long range information defining an embryonic axis that derives from a morphogen gradient. There can be short range information that coordinates the polarity of neighbouring cells. We need to understand the nature of these types of polarising information and ask how they are sent and received. One approach is to try to identify the genes needed in sending cells and discriminate them from those needed in the receiving cells. To do this genetic mosaics have proved essential, both in *Drosophila* (see for example Gubb and Garcia-Bellido, 1982; Vinson and Adler, 1987; Taylor et al., 1998; Wolff and Rubin, 1998; Chae et al., 1999; Usui et al., 1999; Casal et al., 2002; Strutt and Strutt, 2002; Yang et al., 2002) and in vertebrates (see for example Jessen et al., 2002; Wada et al., 2005, 2006; Devenport and Fuchs, 2008).

## How many genetic pathways in PCP?

In *Drosophila*, spontaneous mutations that cause bristle disorientation such as *frizzled* (*fz*) (Gubb and Garcia-Bellido, 1982; Adler et al., 1987; Vinson and Adler, 1987), *dachsous* (*ds*) (Adler et al., 1998) and *fat* (*ft*) (Casal et al., 2002; Strutt and Strutt, 2002; Yang et al., 2002) were later augmented by genes discovered through dedicated screens, such as *starry night* — *stan*, also known as *flamingo* — (Chae et al., 1999; Usui et al., 1999) and *Van Gogh* — *Vang,* also known as *strabismus* — (Taylor et al., 1998; Wolff and Rubin, 1998). Studies on these genes have established that there are (at least) two sets of genes that drive PCP:

1. the Ds/Ft system which incorporates at least two other key proteins, Dachs and Four-jointed (for a review see Thomas and Strutt, 2012).
2. the Fz/Stan system that incorporates at least one other key protein, Vang (for a review see Adler, 2012).

In many recent papers the number of independent PCP systems (one or two?), a central issue, is usually described simply as controversial and left unresolved. In our opinion the one-pathway hypothesis, that the proteins of the Ds/Ft system act upstream to drive the Fz/Stan system, is justified more by tradition than by logic. The arguments for this hypothesis are weak and the experimental evidence flawed — discussed in Lawrence et al. (2007). Against this hypothesis there is one piece of evidence that trumps all the other less persuasive arguments that can be marshalled on both sides: this is the demonstration that, in the absence of a functioning Fz/Stan system, cells containing different amounts of Ds or Ft can polarise responding cells effectively and *in vivo* (Casal et al., 2006). Thus the Ds/Ft system can act very well without the Fz/Stan system. However others do not agree with this interpretation and have argued that the Stan mutant genotype we used to inactivate the Fz/Stan system might not do so sufficiently (see Axelrod, 2009; Peng and Axelrod, 2012). We find that argument feeble, for two reasons: (1) the Ds/Ft signal can still repolarise cells of this Stan mutant genotype even when, in addition, Fz is completely removed from the fly and (2) the same Stan mutant genotype we used completely blocks the ability of *fz*^—^ cells or cells that over-express *fz* to polarise the responding cells *in vivo* (Casal et al., 2006). And there is more evidence in favour of the independence of the two systems that comes from the adult abdomen. Although in the *A* compartment the orientations of the Ds/Ft and Fz/Stan systems are concordant (as they should be if they were part of one pathway), they oppose each other in the *P* compartment (see below).

Others maintain that, since our two-pathway conclusion depends on results in the abdomen, it might not apply to other organs such as eye and wing. This opinion could be correct, but it makes little sense to us as fundamental mechanisms are normally conserved from organ to organ and usually from species to species. Indeed, there is some evidence for two pathways acting in parallel in the eye (Strutt and Strutt, 2002) and in the wing (Strutt and Strutt, 2002; Brittle et al., 2012)

Although the two PCP systems are able to polarise cells independently, both systems have elements of design in common, for example both depend on intercellular bridges: for the Ds/Ft system these are Ds–Ft heterodimers while for the Fz/Stan system these are based on Stan–Stan homodimers. Both systems rely on primary long-range gradients of secreted morphogens to drive secondary gradients that polarise the cells more directly. In the Ds/Ft system, the secondary gradients of Ds and Four-jointed activity regulate the disposition and activity of the heterodimeric bridges (Simon, 2004; Casal et al., 2006; Matakatsu and Blair, 2006; Simon et al., 2010; Brittle et al., 2012). When the Ds and Fj gradients were substituted by uniform concentrations of the two proteins, the orderly orientation of ommatidia was lost. Also, a reverse gradient of Ds and Fj reversed the orientation of ommatidia (Simon, 2004). However, equivalent experiments in the wing did not disturb the polarity of hairs (Simon, 2004); but this inconsistency may be explained by the Fz/Stan system which was intact in these experiments and might have compensated for loss or change of the Ds and Fj gradients. For the Fz/Stan system there is some evidence that the secondary gradient is of Fz activity (Adler et al., 1997; Lawrence et al., 2004); but there are mixed opinions about this (Peng and Axelrod, 2012). Adler and colleagues offered the first evidence that polarity of hairs might depend on a gradient of Fz activity; when a reversed gradient of Fz was produced artificially the hairs pointed in the opposite direction (Adler et al., 1997).

It is thought that a slope of the Fz activity gradient is read by asymmetric bridges (Lawrence et al., 2004, 2008b). Ideas about these bridges and how they function are undergoing frequent revision as evidence is gathered; they were envisaged as Stan–Stan homodimers that are linked to Fz in one cell and Vang in the other (see Strutt and Strutt, 2009). Recently we have presented evidence that each bridge is a Stan–Stan homodimer that associates with Fz only on one side, allowing the bridges to compare the amount of Fz in neighbouring cells. Vang acts only to assist this process and is not an essential part of the bridge itself (Struhl et al., 2012) — a hypothesis consistent with earlier results (Strutt and Strutt, 2008).

## PCP, axes, compartments, morphogens and gradients

Since organisms are largely made of epithelia, two axes usually suffice to specify the cells. For example the limbs of *Drosophila* are cylindrical, they have a proximodistal axis; they are divided longitudinally into anterior (*A*) and posterior (*P*) compartments by cell lineage. The bristles and hairs on the leg point distally. The wing blade is topographically like a leg squashed flat, with the boundary between *A* and *P* compartments running in the proximodistal axis along the middle of the wing (reviewed in Blair (1995)). Each cell produces one hair that points distally, that is parallel to the *A*/*P* compartment boundary. In the trunk, a series of metameres, defined initially as parasegments, each become later subdivided into a *P* and an *A* compartment. In contrast to the wing, the bristles and hairs in the insect thorax and abdomen point posteriorly, that is perpendicular to the *A*/*P* borders. In the eye the situation is again different. The eye consists of multicellular ommatidia and is derived from the *A* compartment of the antennal segment (Morata and Lawrence, 1978). There is also a Hedgehog-dependent boundary that advances steadily across the eye and drives development of the ommatidia (Rogers et al., 2005). The eye is divided by an equator with the ommatidia being oriented at right angles to that equator; above and below the equator the ommatidia have opposite chiral forms.

**Fig. 1:**
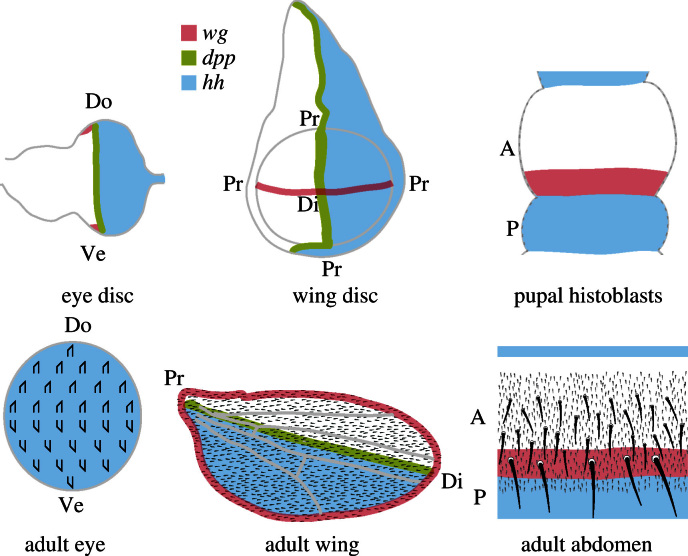
Summary of the axes and morphogens of the eye, wing and abdomen of *Drosophila*, the main organs used to study PCP. The colours indicate the zones of expression of genes encoding the three morphogens, showing their radically different dispositions. Note the structures that indicate PCP: ommatidia in the eye, hairs of the wing and hairs and bristles of the abdomen. Note that while the wing hairs are aligned parallel to the *A*/*P* axis, the abdominal hairs and bristles are orthogonal to the same axis. The ommatidia are aligned along the *A*/*P* axis but have two chiral forms depending if they are dorsal or ventral in the disc. Do=dorsal, Di=distal, Pr=proximal, Ve=ventral.

Even though these three model systems are so different, they depend on a common set of morphogens to pattern the cells. Thus, in the wing, the main morphogens are Hedgehog (Hh), Decapentaplegic (Dpp) and Wingless in both compartments (Blair, 1995). In the anteroposterior axis of the dorsal abdomen, Hh operates in the *A* compartment and, probably, Wingless in the *P* while the ventral abdomen deploys Dpp (Struhl et al., 1997a, 1997b; Lawrence et al., 2002). In the mediolateral/dorsoventral axis of the adult abdomen Hh defines expression of Dpp and Wingless which interact with each other to mark out tergites, pleura and sternites (Kopp et al., 1999). In the eye, Dpp, Hh and Wingless are again used (see Tsachaki and Sprecher, 2012 for a review). Thus, in these systems, while overlapping sets of morphogens are deployed, the orientation of the morphogen gradients and the orientation of structures differ (Fig. 1). Nevertheless it is a fair assumption that, in these different systems, the downstream mechanisms of PCP used to polarise cells are largely conserved. But is this assumption correct? This answer to this question is unknown.

## Using genetic mosaics to map polarity gradients

**Fig. 2:**
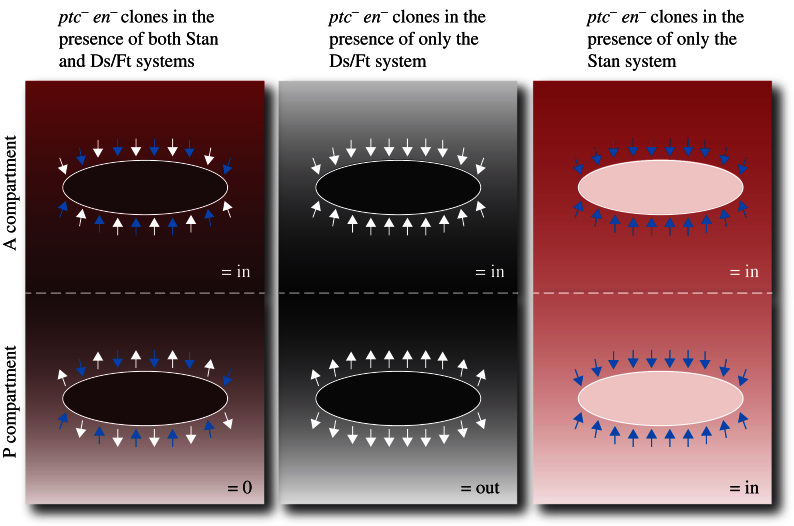
The polarity effects of *ptc*^—^*en*^—^ clones. This model is puzzling, but it illustrates some important facts and principles about the two PCP systems operating in the *Drosophila* abdomen. Anterior is at the top. In the wildtype, we envisage two gradient systems, one (Ds activity) from the Ds/Ft system shown in grey and one (Fz activity) from the Fz/Stan system shown in red. The Ds/Ft gradient peaks at the *A*/*P* compartment border (dashed line) and runs downwards from there in both anterior and posterior directions. The Fz gradient may have a more complex topography than shown but most likely peaks at the front of the *A* compartment, declining posteriorwards from there to the back or middle of the *P* compartment. In both the *A* and *P* compartments the hairs point down the Fz activity gradient but the two compartments read the Ds gradient with opposite sign, pointing ***up*** the Ds slope in the *A* compartment, and ***down*** the Ds slope in the *P* (Casal et al., 2002). In the *A* compartment the slopes of both the Fz and Ds activity gradients act together to point the hairs backwards while in the *P* compartment the two gradients act with opposite sign. In both the *A* and the *P* compartments, clones that lack the *ptc* and *en* genes make cuticle that shows the identity of cells that are located in the wildtype just anterior to the *A*/*P* compartment boundary; within these clones the levels of the gradients are indicated by the colour intensities. These cell identities correspond to high levels of Ds activity (dark grey) and medium levels of Fz activity (pale pink). The arrows indicate the directions of the local slopes of Ds activity (white) and Fz activity (blue) near the clones; in the *A* compartment these local slopes orient hairs near the clones down the Fz activity gradient and up the Ds activity gradient. However in the *P* compartment the *P* cells orient their hairs down the Ds slope (Casal et al., 2002). Thus in the *P* compartment of the wildtype, hairs near *ptc*^—^*en*^—^ clones are subject to two opposing influences; explaining perhaps why these clones have no consistent effect on the cells around them.

In the adult abdomen, the hairs and bristles point backwards in both the *A* and the *P* compartments, but the effects of clones on polarity of neighbouring cells argue that the two gradient systems that drive PCP have different forms. The polarising gradient of Ds activity (shown grey in Fig. 2) peaks at or near the *A*/*P* border and probably declines from there in both directions, giving opposing slopes in the *A* and the *P* compartment (Casal et al., 2002). This raises no problems with respect to the chain of metameres, as these gradients could be continuous across the compartment borders. However the behaviour of clones affecting the Fz/Stan system argue the slope of Fz activity is inclined in the same orientation in the *A* and in the *P* compartment. If this is so it raises a known unknown: how and where does the Fz activity gradient (red in Fig. 2) repeat from metamere to metamere?

*ptc*^—^ *en*^—^ clones made in the *A* compartment of the abdomen help make the case that Hh signalling drives both PCP systems to produce gradients of the two polarising signals. Cells that lack Ptc have high levels of Hh signalling so that they develop as if they were located at the back of the *A* compartment (Struhl et al., 1997b); there we envisage the Ds concentration to be high and Fz concentration low (Casal et al., 2002, 2006). Accordingly, when such clones are made almost anywhere within the *A* compartment (but not right at the back where their cells resemble the surrounding ones) they change the polarity of wildtype cells near to them and behave as if they had a high Ds activity and lacked Fz. In the *P* compartment, given the way we imagine the gradients slope, we expect the *ptc*^—^ *en*^—^ clones to try to affect the nearby hairs but in two mutually opposing directions (blue arrows for the Fz/Stan system and white arrows for the Ds/Ft system in Fig. 2). Our unpublished results on the *P* compartment confirm this view; in a wildtype background the clones do not repolarise the surroundings, but in a *ds*^—^ background (inactivating the Ds/Ft system) *ptc*^—^ *en*^—^ clones in the *P* compartment behave like *fz*^—^ clones and cause hairs to point inwards. Accordingly, in a *stan*^—^ background (inactivating the Fz/Stan system) *ptc*^—^ *en*^—^ clones in the *P* compartment behave like *ds*-expressing clones and cause hairs to point outwards (Fig. 2). Thus the special situation in the *P* compartment highlights a central question: how do the two PCP systems integrate to determine the polarity of a cell in the wildtype? The answer to this question is unknown.

One can investigate this matter further by looking at the polarity of hairs in flies in which one system is knocked out completely. Thus, in the absence of the Fz/Stan system and if the two systems are totally independent, the hair polarity should be a direct read-out of the Ds/Ft gradients. As argued above from clones that affect the Ds/Ft system made in *fz*^—^ flies, the hairs should point posteriorly in the *A* compartment but anteriorly in the *P* compartment (as they do in the larva, see below). But they do not: the hairs in the *P* compartment of *fz*^—^ flies are more or less normal in orientation. We do not understand why; it may be that we are misunderstanding the results we have. Alternatively, this finding might be telling us that there are additional mechanisms, in addition to the two systems we study. And there are other suggestions of additional mechanisms: flies that lack both the Ds/Ft and the Fz/Stan systems (*ds*^—^ *stan*^—^) have largely randomised hair polarity but do reach the adult stage, even though they are dysmorphic and remain stuck in the pupae (Casal et al., 2006). So, if PCP is as central and important as we like to think, with, possibly, input into many cell behaviours including axon growth, cell migration, and orientation of mitosis, then how can these mutant flies develop so far?

Similarly, in the absence of the Ds/Ft system, the hairs and bristles should reveal the underlying polarity effects of the Fz/Stan system working alone. The clones tell us that that hair orientation should be normal, as in both in the *A* and the *P* compartments the hairs point consistently into *fz*^—^ clones. But, in *ds*^—^ flies, hair orientation is not normal: near the middle of the *P* compartment hairs mostly point anteriorly (see Fig. 5 in Casal et al., 2002). The simplest explanation is that the Fz gradient has a low point near the middle of the *P*, with the Fz activity increasing from there both anteriorly and posteriorly. In addition, in both *ds*^—^ and *ft*^—^ flies, over most of the *A* compartment and at the front of the *P*, hair polarity is disorganised and swirly; but we think the cause of this phenotype is special and is discussed with the Hippo pathway below.

## Polarised denticles in the larval abdomen

Studies on the larval abdomen also raise some known unknowns. The orientations of larval denticles are certainly an outcome of PCP, but they are more complex than one might expect. In the third stage larva there are 7 somewhat irregular rows, rows 0 and 1 are made by *P* compartment cells and point forwards, while rows 2, 3, 5 and 6 are made by the *A* compartment and point backwards — row 4 idiosyncratically points forwards. The pattern and orientation of denticles in *stan*^—^ or *fz*^—^ embryos and larvae are almost completely normal, arguing that the Fz/Stan system has only a small input into denticle polarity (Casal et al., 2006; Repiso et al., 2010). But the Ds/Ft system is the major determinant; in its absence all denticles are awry in the larva (Casal et al., 2006; Repiso et al., 2010). Our expectation was that the slopes of Ds and Fj would determine the polarity of the denticles. In the wildtype larva the gradients of the Ds/Ft system in the *A* and the *P* compartments might well oppose each other (as they do in the adult, see above), and accordingly one might expect the denticles of the *P* and the *A* compartments to point in opposite directions. And indeed they do and this applies to rows 0 and 1 (*P*, point forwards) and also for rows 2, 3, 5 and 6 (*A*, point backwards). Further evidence for this model comes when the slopes of Ds are changed by experiment: these new slopes change the denticle orientation accordingly (Repiso et al., 2010). However row 4 is contrary, it belongs to the *A* compartment but points forward. It seems that this row is oriented by a special subroutine (Dilks and DiNardo, 2010). Nevertheless, row 4 raises a known unknown; for which the question is, is there a mandatory link between the sign of the PCP machinery and the final orientation of a cell? That is, can the polarity cues that are produced by the PCP proteins be interpreted differently in different organs — just as appears to happen with row 4? This unknown comes up again in the mammalian inner ear, see below.

## What are the polarising signals in Drosophila and vertebrates?

Virtually all reviews on PCP discuss Wnts and this raises another known unknown: are one or more Wnt genes polarising signals in flies and/or in vertebrates? Answering this question is not easy, partly because of the salesmanship that has distorted the whole field of developmental genetics and is duping us all (Lawrence, 2001b). For example, it has become standard practice to show an effect of some mutation on any organ or property (such as PCP) and then conclude from that effect that the gene in question “regulates” or “mediates” or “controls” that organ or property. While logically true, these vague words allow a large number of genes to be categorised as “PCP genes” while hiding our ignorance of mechanism. These words can even deflect us from asking the key question: what do these genes actually contribute to PCP in the wildtype and *in vivo*? This point about the over-identification of PCP genes has also been made by others (e.g., Wang and Nathans, 2007; Gao, 2012).

But how to identify the true polarising signals? To illustrate we can discuss the fly abdomen again: in the *A* compartment do we judge the primary morphogen (Hh) to be a polarising signal? Yes and no. Results argue that the gradient of Hh is upstream and, like other morphogens, it affects both pattern (the arrangement of cells of different types along the gradient axis) and PCP. But for PCP it acts indirectly; there is evidence that it orients a secondary gradient that is immediately (that is “directly”) responsible for orienting the Fz/Stan system of PCP. This secondary gradient is probably a gradient of Fz activity (Adler et al., 1997; Lawrence et al., 2004; Struhl et al., 2012). The key point is made by evidence that the cells being polarised ***are not reading*** the gradient of Hh, but ***are comparing*** the activity of Fz between neighbouring cells. Thus while it remains correct to say Hh “regulates” or “mediates” or “controls” PCP, Hh is not the polarising signal, but Fz activity is.

We have tried to identify one or more Wnts that might be polarising signals in the *Drosophila* abdomen. There are 7 Wnt genes in the fly genome and we have used different tests to see if any have a direct role in PCP. One assay is to remove the Wnt gene in question from a clone of cells and look for effects on polarity (e.g., Chen et al., 2008); however this is a poor test as Wnt proteins can spread into the clone and rescue any effect. Even large *wg*^—^ clones in the wing often develop normally (e.g., Baker, 1988), and noone has argued from this that Wg is immaterial to the wing. *A* better test is to remove a Wnt gene from within a clone that is constituitively active for Hh signalling, and see if the loss of that Wnt blocks the repolarisation that the clone normally induces in nearby cells. This assay was used to test most Wnts including Wg and the answers were negative. We also tried to overexpress the Wnts in clones of cells to see if they can repolarise the surrounding wildtype cells, but there was again no effect (Lawrence et al., 2002). We later even removed the Ds/Ft system from the genetic background to sharpen this test (Casal et al., 2006); however none of these experiments detected any significant repolarisation, suggesting that none of these Wnts act as polarising signals, at least in the *A* compartment. There was one apparent exception, but that concerned the *P* compartments; here removal of a Wnt receptor, Arrow, reoriented cells. However our tentative explanation was that, in the wildtype, Wg acts in the *P* compartments rather as Hh does in the *A* compartments (i.e., as a morphogen) and orients PCP indirectly, possibly via setting up a gradient of Fz activity (Lawrence et al., 2002). So at least for the moment we can conclude that the evidence is against Wnts being the polarising signals in insects.

Now if we turn to the vertebrates particularly to convergent extension: in most or all of the experiments touching on vertebrate Wnts and polarity (there are hundreds of papers) it is still not clear if the Wnt being tested is acting like a morphogen (i.e., having an indirect effect on PCP as well as other effects) or more directly as a polarising signal that is specific for PCP and actually read by the PCP mechanisms in cells. If we take the paradigm case of Wnt11 in convergent extension in *Xenopus* (Heisenberg et al., 2000; Wallingford et al., 2002), do the experiments distinguish between a direct and indirect effect? Do the experiments argue that the cells compare levels of Wnt11 activity between neighbouring cells, to determine the slope of a Wnt11 gradient and thereby to orient their planar polarity? We judge the answer to these questions to be no and, generally, conclude there is doubt about whether Wnts are the ultimate polarising signals in vertebrate PCP, even though they are often claimed to be. To resolve the doubt might require different approaches as used in *Drosophila*. For example when Wnt11-expressing cells were grafted between nascent somites the muscle fibres around the graft are reoriented (Gros et al., 2009) but it is not clear from this whether the Wnt11 is a polarising signal by the definition given above — it could still be acting as a morphogen and producing a local gradient of another molecule. Thus it is even possible that the most immediate polarising signal for the Fz/Stan system of PCP in vertebrates is not a Wnt but, as some of us favour for *Drosophila*, a gradient of Fz activity (Dabdoub et al., 2003; Gao, 2012).

## The asymmetric localisation of PCP proteins: Is this a cause or an effect of PCP?

This question constitutes another known unknown. That PCP proteins are localised asymmetrically in the cell was discovered by Axelrod (2001) for Dishevelled, and by Strutt (2001) for Fz. The discovery has since been widened to many PCP proteins (Strutt and Strutt, 2009) but its function is still not clear. It is very likely that preferential localisation is functional and indeed it is a feature of most models for the mechanisms of PCP, but that conclusion is compromised by several observations:

i. in *pk*^—^ flies, localisation of several key proteins is apparently abolished (Strutt, 2001) and yet the ability of these cells to transmit polarity signals is intact (Lawrence et al., 2004; Strutt and Strutt, 2007). Not only intact, it is actually enhanced (Adler et al., 2000; Lawrence et al., 2004) and we offer an explanation for this: the function of Pk in the wildtype may be concerned with ***intracellular*** and asymmetric localisation of PCP proteins and not with cellular interaction. Thus, in the absence of Pk, cells will lack a robust asymmetry and any residual polarity will depend on ***intercellular*** protein interactions; such cells will be more easily repolarised by changing the amounts of Fz in neighbouring cells.
ii. Cells show this protein assymmetry clearly only at limited times (for example in the *Drosophila* wing, only shortly before the wing hairs extend) yet in our opinion it is likely that the cells are polarised for much longer periods, including when they do not demonstrate protein asymmetry. An example is the *Drosophila* blastoderm mentioned earlier. One explanation that gets around the concerns raised in points 1 and 2 is to suggest that the proteins are indeed usually localised asymmetrically in the cell; but weakly so that it the asymmetry is difficult to detect (Aigouy et al., 2010).
iii. In the vertebrate inner ear, there is no consistent correlation between the localisation of proteins and the polarity of the stereocilia. For example, in the utricle, the Pk and Fz proteins are localised assymmetrically in the cell but their localisations do not correlate with the orientations of the bundles of stereocilia (Deans et al., 2007). Also in the cochlea, Vangl2 and Fz are localised on the same side of the cell (Wang et al., 2006), whereas in the fly it is considered central to function that these proteins are preferentially localised on opposite sides of the cell.

## What is the wildtype function of the Hippo pathway?

Many authors investigate and discuss the relationship between the Hippo pathway and PCP. The Hippo pathway is advertised as a tumour suppressor pathway or as a pathway responsible for determining growth, or as a pathway determining organ shape and size, or as involved in PCP, or as having all of these properties (Harvey and Tapon, 2007; Zhao et al., 2011). By these means Hippo pathway workers claim membership of at least four fashionable fields. But, unfortunately, there is only scant evidence to support these claims. In order to achieve a realistic understanding of the Hippo pathway, we believe the first important question should be: what does Hippo pathway do in the wildtype fly? The answer to this question is unknown. We can start to answer it by accepting that the Fat gene is connected to the Hippo pathway. For example, clones of cells lacking *ft* activate *expanded* and *Diap-1*, downstream targets of the Hippo pathway. Yet Fat is also a founding member of the Ds/Ft system for PCP. Thus it is clear that the Hippo pathway and PCP are linked through Fat, but how? There are two pieces of hard information that argue in different directions. One, using an *in vivo* cellular assay*,* changes in PCP are initiated when only the ***extracellular*** parts of either Fat and Ds are expressed (Casal et al., 2006). Two, the whorly polarity characteristic of *ft*^—^ or *ds*^—^ flies can be rescued by expression of the ***intracellular*** domain of Fat (Matakatsu and Blair, 2006). So, how can the polarity of a cell be determined by the distribution of extracellular domains of Fat and yet a whorly polarity phenotype be rescued by the intracellular domain of Fat? The resolution we suggest is that the intra and extracellular domains of Fat act in different processes in the wildtype. The extracellular portion would be engaged with the extracellular domain of Ds in adjacent cells to form intercellular bridges that exchange polarity information (Ma et al., 2003; Matakatsu and Blair, 2004; Casal et al., 2006); so that indeed, in PCP, Ds and Fat are essentially equivalent. The intracellular portion of Fat could act via Dachs and Warts to promote phosphorylation of Yorkie, thereby reducing the import of Yorkie into the nucleus (Huang et al., 2005; Oh and Irvine, 2008). Accordingly, in *ft*^—^ or *ds*^—^ flies, where Fat is either not present or does not accumulate normally at the membrane (Ma et al., 2003), Yorkie would enter the nucleus and and somewhat disorganised growth would follow. We cannot explain how this disorganisation and growth could cause a whorly phenotype, but even so it is our hypothesis that it does and does so without interfering with the central mechanisms of PCP. This hypothesis helps us understand why the whorly phenotype is rescued by uniform expression of the intracellular domain of Fat and not by the intracellular domain of Dachsous (Matakatsu and Blair, 2006) — in accord with the conclusion that Ds and Ft are ***not*** equivalent with respect to their interaction with the Hippo pathway. Fat is increasingly being seen as having several outputs, some acting through the Hippo pathway and some not (Matakatsu and Blair, 2012; Marcinkevicius and Zallen, 2013; Pan et al., 2013) — and indeed, there is much yet to understand about the wildtype function of Fat.

Explaining why the two domains of Fat appear to have two separate functions is important. While it is now fairly clear how Fat acts in PCP, its relevance to “growth” remains mysterious. As we have seen, one firm piece of evidence that Fat does impact on growth is that clones of cells mutant for Fat grow excessively as well as being somewhat disorganised. However it is also relevant that entirely *ds*^—^ or *ft*^—^ flies, although somewhat dysmorphic, are not very much larger than wildtype flies; it follows that extra growth is not a necessary consequence of lacking the Ds/Ft system. To try to make some sense of these contrary pieces of evidence we have speculated that, in the wildtype fly, the Ds/Ft pathway may be involved in dimension measurement: a basic premise would be that the local steepness of the polarising signal for Ds/Ft system would normally correlate with the length of an organ in the gradient axis. If some measure of this steepness could be fed back to the single cell, it could, in effect, tell that cell how long the organ is in the measured axis and increase or decrease its propensity to divide. In this way the steepness would regulate growth. This speculation is largely based on model building, on experiments on the cockroach limb (Bohn, 1974) but crucially on mosaic experiments (Rogulja et al., 2008; Willecke et al., 2008) which show that abrupt differences (i.e., a steep local gradient) in amounts of Ds, Ft or Fj across the interfaces between groups of cells drives both local polarity changes and local and extra cell divisions — this evidence is discussed elsewhere (Lawrence et al., 2008a). If this hypothesis were allowed, one could begin to see how the Hippo pathway might act in the wildtype to drive and/or block growth depending on inputs carried by the intracellular domain of Fat. How much Fat is in localised in the membrane of a cell should depend on the disposition of Ds in neighbouring cells and thus Ds would also help link growth and PCP.

However there is a different body of evidence that organisation of growth, specifically in the wing, may not depend on tissue-wide gradients of Ds and/or Fj. In this model Wingless, acting as a morphogen, sponsors an interaction between Ds and Ft that spreads as a wave, recruiting cells to wing and driving growth of these cells by acting via Dachs, Warts and Yorkie on the wing gene, Vestigial (Zecca and Struhl, 2010). The contrasts between these different models and the lack of any coherence between them illustrates how much we have still to learn about two intriguing and important known unknowns: what limits growth to define organ size and what does the Hippo pathway do in the wildtype?

“Wir müssen wissen — wir werden wissen!” David Hilbert (german mathematician) September 8th 1930.
